# Pontine Tegmental Infarction With Cerebellar Involvement Mimicking Lateral Medullary Syndrome: A Clinico-Radiological Paradox

**DOI:** 10.7759/cureus.110367

**Published:** 2026-06-06

**Authors:** Abhishek Chanda, Dipra Dattasarma, Souvik Sen, Priyodeep Saha, Faisal Sarfaraz

**Affiliations:** 1 Internal Medicine, KPC Medical College and Hospital, Kolkata, IND

**Keywords:** brainstem stroke, clinico-radiological dissociation, lateral medullary syndrome, pontine infarction, posterior circulation ischemia

## Abstract

Lateral medullary syndrome (LMS), or Wallenberg syndrome, typically arises from ischemia of the dorsolateral medulla and is characterized by crossed sensory deficits, ipsilateral cranial nerve involvement, cerebellar ataxia, and Horner’s syndrome. We report a 66-year-old hypertensive female who presented with classical clinical features suggestive of LMS, including ipsilateral facial sensory loss, contralateral body sensory impairment, palatal weakness, ipsilateral Horner’s syndrome, and cerebellar signs. However, diffusion-weighted magnetic resonance imaging revealed a predominantly paramedian pontine tegmental infarction with associated right cerebellar involvement, without evidence of medullary ischemia. This clinico-radiological dissociation can be explained by the involvement of shared neuroanatomical pathways, such as the spinothalamic tract, spinal trigeminal nucleus, and descending sympathetic fibers, which extend into the pontine tegmentum, thereby mimicking LMS. This case highlights the limitations of clinical localization in posterior circulation stroke and emphasizes the importance of neuroimaging in accurately identifying lesion sites when brainstem syndromes present with overlapping features.

## Introduction

The pons, a critical component of the brainstem connecting the midbrain and medulla, derives its vascular supply predominantly from the paramedian branches of the basilar artery, along with contributions from the anterior inferior cerebellar artery and, in its rostral portion, the superior cerebellar artery [1]. Ischemia involving these territories produces a spectrum of pontine syndromes depending on the rostrocaudal level and structures affected [1,2]. Classically, medial pontine infarctions due to paramedian branch occlusion present with contralateral motor deficits, medial lemniscus involvement, cranial nerve deficits, and variable ataxia or gaze abnormalities [3,4].

In contrast, lateral medullary syndrome (LMS) typically results from occlusion of the posterior inferior cerebellar artery or vertebral artery and is characterized by ipsilateral facial sensory loss, contralateral body pain and temperature deficits, cerebellar ataxia, nucleus ambiguus involvement, and Horner’s syndrome [2,4]. Although posterior circulation strokes account for approximately 20-25% of all ischemic strokes, LMS itself represents only a small proportion (~2-3%), highlighting its relative rarity [4,5].

Despite these classical clinico-anatomical correlations, lesions within the pontine tegmentum may mimic LMS due to the longitudinal extension of shared neural pathways, including the spinothalamic tract, spinal trigeminal nucleus, and descending sympathetic fibers [6-8]. Reports of pontine infarction presenting as LMS are exceedingly rare and largely limited to isolated case reports, underscoring the potential for misleading clinical localization and the critical role of neuroimaging in such cases [9-12].

## Case presentation

A 66-year-old hypertensive female presented approximately 48 hours after symptom onset with severe vertigo, recurrent vomiting, and inability to maintain a sitting posture. She initially received primary symptomatic care at a peripheral health center, including intravenous fluids and antiemetics, and was subsequently referred to our institution for further evaluation. No neuroimaging or thrombolytic therapy was performed prior to transfer. She was a known hypertensive on irregular antihypertensive therapy. There was no known history of diabetes mellitus, smoking, atrial fibrillation, prior cerebrovascular accident, ischemic heart disease, or chronic kidney disease. No prior antiplatelet or anticoagulant use was reported.

On admission, the patient was conscious, alert, and cooperative, with preserved higher mental functions; her blood pressure was 160/90 mmHg, pulse rate was 96 beats/minute and regular, and oxygen saturation was 94% on room air. Cranial nerve examination revealed left-sided lower motor neuron facial weakness involving both upper and lower facial musculature, consistent with House-Brackmann grade IV facial paresis, impaired pain and temperature sensation, along with reduced fine touch sensation, over the left half of the face. Palatal movement was impaired on the left side. Mild dysarthria was present. Gag reflex was diminished on the left side. No overt aspiration or respiratory compromise was observed during hospitalization. The tongue showed no deviation or atrophy; however, tongue movements appeared slow and stiff without fasciculations, suggesting possible supranuclear corticobulbar involvement rather than lower motor neuron hypoglossal dysfunction.

Autonomic examination demonstrated partial left-sided ptosis with miosis and anisocoria, more pronounced in dim illumination, consistent with left-sided Horner’s syndrome. Apparent enophthalmos was noted clinically without radiological confirmation. Sensory examination of the body revealed impaired pain and temperature sensation over the right half of the body, consistent with spinothalamic tract involvement.

Motor strength was preserved in all four limbs; however, deep tendon reflexes were brisk and plantar responses were bilaterally extensor. Tone was normal. The patient exhibited a tendency to fall toward the left side, with bilateral horizontal nystagmus (right greater than left). Finger-nose testing was impaired on the right side, indicating cerebellar involvement. Ocular movements were full, and diplopia was absent. Bladder and bowel functions were normal.

Although the constellation of findings, as summarized in Table 1, the crossed sensory deficits, Horner’s syndrome, palatal involvement, vertigo, and cerebellar signs strongly suggested LMS clinically, the presence of bilateral extensor plantar responses, corticobulbar features, and lower motor neuron facial weakness were atypical and raised the possibility of pontine involvement.

**Table 1:** Summary of clinical findings HMF: higher mental function; LMN: lower motor neuron; UMN: upper motor neuron

Domain	Finding	Side	Interpretation / Localization
Demographics	66-year-old female, hypertensive	—	Vascular risk factor (stroke predisposition)
Presenting Symptoms	Vertigo, vomiting, inability to sit	—	Suggests vestibular/cerebellar involvement
Consciousness & Cognition	Alert, normal HMF	—	Cortical functions preserved
Cranial Nerve VII	LMN facial palsy	Left	Pontine (facial nucleus/fascicle involvement)
Cranial Nerve V (Sensory)	Loss of pinprick & fine touch	Left face	Spinal trigeminal tract involvement
Cranial Nerve IX/X	Impaired palatal movement	Left	Possible corticobulbar pathway involvement producing LMS-like bulbar features
Cranial Nerve XII	Slow, stiff tongue movements without atrophy or fasciculations	Bilateral	UMN involvement (corticobulbar tract)
Sympathetic Pathway	Left-sided ptosis, miosis, and anisocoria consistent with Horner syndrome	Left > Right	Descending sympathetic tract involvement
Sensory (Body)	Impaired pain and temperature sensation	Right body	Spinothalamic tract involvement (crossed finding)
Motor System	Normal power	—	Corticospinal tract relatively spared (early/partial)
Reflexes	Hyperreflexia + bilateral extensor plantar	Bilateral	UMN involvement
Tone	Normal	—	No established pyramidal rigidity yet
Cerebellar Signs	Finger-nose test positive	Right	Ipsilateral cerebellar hemisphere involvement
Posture	Falls/leans to left	Left	Vestibulocerebellar dysfunction
Nystagmus	Horizontal, bilateral (R > L)	—	Brainstem/cerebellar involvement
Ocular Movements	Normal, no diplopia	—	6th nerve spared → atypical for pontine lesion
Autonomic	Bladder/bowel normal	—	No spinal/autonomic disruption

Her diffusion-weighted MRI brain demonstrated acute non-hemorrhagic infarction involving the predominantly paramedian pontine tegmental region, along with an acute infarct in the right cerebellar hemisphere, likely within the posterior circulation territory (Figure 1).

**Figure 1:**
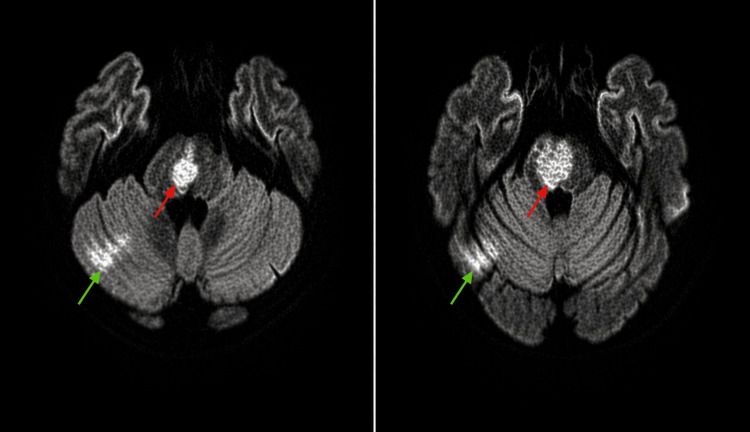
Diffusion-weighted MRI brain demonstrating acute paramedian pontine tegmental infarction (red arrow) with associated acute right cerebellar infarction (green arrow)

MR angiography demonstrated non-visualization of the right vertebral artery and bilateral P1 segments of the posterior cerebral arteries, along with a hypoplastic left vertebral artery and basilar artery (Figure 2). These findings may reflect underlying hypoplasia, reduced flow, thrombotic occlusion, or flow-related imaging artifact. No confirmatory CT angiography or Doppler imaging was available.

**Figure 2:**
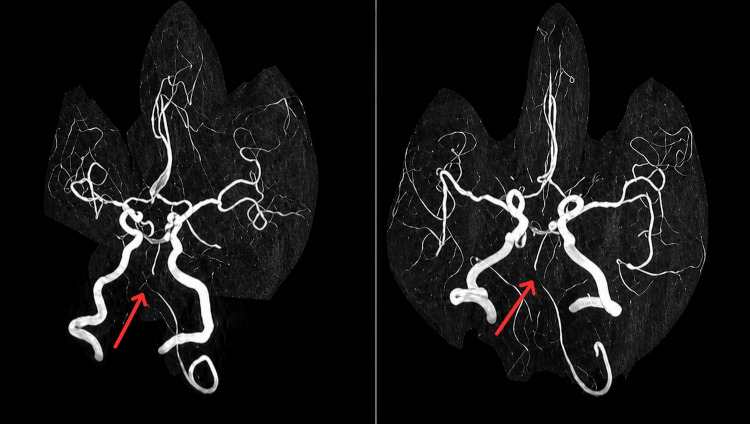
Time-of-flight MR angiography demonstrating non-visualization of the right vertebral artery (red arrow) The finding may represent vascular occlusion, congenital hypoplasia, reduced flow, or flow-related imaging limitation.

Radiological findings are summarized in Table 2.

**Table 2:** Summary of radiological findings PCA: posterior cerebral artery

Modality	Finding	Interpretation
MRI Brain (DWI)	Acute non-hemorrhagic paramedian pontine tegmental infarction + right cerebellar hemisphere infarction	Confirms pontine infarct with cerebellar involvement
MR Angiography	Non-visualization of right vertebral artery	Suggestive of vertebrobasilar flow abnormality; differential considerations include hypoplasia, reduced flow, or vascular occlusion
	Non-visualization of bilateral P1 PCA segments	May reflect altered posterior circulation flow; clinical significance uncertain
	Hypoplastic left vertebral and basilar artery	Potential contributor to reduced posterior circulation reserve

Magnetic resonance imaging with diffusion-weighted sequences and MR angiography revised the initial clinical impression of LMS to posterior circulation stroke due to pontine and cerebellar infarction with underlying vertebrobasilar pathology, as described in Table 3. As the patient presented approximately 48 hours after symptom onset, she was not eligible for intravenous thrombolysis or mechanical thrombectomy. She was managed conservatively with dual antiplatelet therapy (aspirin 75 mg plus clopidogrel 75 mg daily), high-intensity statin therapy (atorvastatin 80 mg daily), blood pressure optimization, hydration, swallowing assessment, and supportive neurorehabilitative care, including physiotherapy and gait assistance. At discharge, vertigo and truncal instability had improved significantly, and the patient was able to sit with minimal support. Residual mild gait ataxia persisted. The modified Rankin Scale score at discharge was 2. She was advised for regular follow-up.

**Table 3:** Explanation of the clinical syndrome LMS: lateral medullary syndrome

Clinical Syndrome Suggested	Actual Lesion	Explanation
Lateral medullary syndrome (Wallenberg)	Acute paramedian pontine tegmental infarction	Due to shared pathways in the pontine tegmentum
Ipsilateral facial sensory loss + contralateral body loss	Pontine involvement of trigeminal + spinothalamic tracts	Mimics the LMS pattern
Horner’s syndrome	Descending sympathetic fiber involvement	Extends beyond the medulla into the pons
Ataxia	Cerebellar infarct + peduncle involvement	Explains truncal and limb incoordination

## Discussion

Our case illustrates a striking clinico-radiological dissociation, wherein a classical phenotype of LMS was produced by paramedian pontine tegmental infarction with associated cerebellar involvement. Traditionally, LMS results from ischemia of the dorsolateral medulla, most commonly due to occlusion of the posterior inferior cerebellar artery or vertebral artery, and is characterized by ipsilateral facial sensory loss, contralateral body pain and temperature deficits, nucleus ambiguus involvement, cerebellar ataxia, and Horner’s syndrome [1,4]. The present case is particularly unusual because of the combination of predominant pontine tegmental localization, associated cerebellar infarction, vertebrobasilar vascular abnormalities on angiography, crossed sensory findings, Horner’s syndrome, bulbar symptoms, and cerebellar signs occurring without radiological evidence of medullary infarction. This combination produced a close clinical mimic of LMS despite a non-medullary lesion location.

However, the brainstem contains longitudinally oriented neural pathways that traverse both the medulla and pons, thereby permitting overlap in clinical syndromes [3]. In this case, involvement of the spinal trigeminal pathways, spinothalamic tract, and descending hypothalamo-spinal sympathetic fibers within the pontine tegmentum likely explains the presence of crossed sensory deficits and Horner’s syndrome despite the absence of a medullary lesion [1,8]. Descending sympathetic fibers traverse the dorsolateral brainstem and may be affected by pontine tegmental infarction, thereby producing partial Horner’s syndrome without medullary involvement. Such extension of these pathways into the dorsolateral pons has been described as a key anatomical substrate for false localization in posterior circulation strokes [2,4,8].

Additionally, cerebellar signs, including ipsilateral limb ataxia and truncal instability, can be attributed to the involvement of cerebellar connections, likely related to the associated cerebellar hemispheric infarction and pontine vestibulocerebellar pathway involvement, further reinforcing the LMS-like clinical picture [7,10,11]. Although finger-nose incoordination was more prominent on the right side, the tendency to fall toward the left may reflect associated vestibulocerebellar pathway dysfunction within the brainstem rather than isolated hemispheric cerebellar involvement alone.

Certain atypical features in this case warrant specific consideration. The absence of abducens nerve involvement suggests sparing of the ventral pontine structures where the sixth nerve nucleus and fascicles are located, indicating a predominantly tegmental rather than basilar pontine lesion [1,5]. Impaired palatal movement, although classically associated with nucleus ambiguus involvement in medullary lesions, may alternatively reflect supranuclear corticobulbar pathway disruption in pontine tegmental infarction [2,6]. Since no medullary lesion was identified radiologically, a corticobulbar mechanism appears more anatomically plausible in the present case. Similarly, the presence of slow and stiff tongue movements without atrophy or fasciculations supports corticobulbar tract involvement rather than a lower motor neuron hypoglossal lesion [6].

Collectively, these findings underscore that the clinical phenotype of LMS can arise from lesions outside the medulla due to interruption of shared and overlapping neural networks within the brainstem [1,8]. This case highlights the limitations of relying solely on clinical localization and emphasizes the indispensable role of advanced neuroimaging in accurately identifying lesion sites in posterior circulation stroke, where anatomical-functional correlations may be complex and non-intuitive [9,12].

## Conclusions

This case demonstrates that posterior circulation strokes can present with deceptively classical clinical syndromes that do not accurately reflect the site of infarction. It emphasizes the need to maintain diagnostic caution when evaluating brainstem lesions based solely on clinical findings. Early use of MRI and MR angiography is essential for precise localization and guiding appropriate management. In our patient, conservative stroke management, including antiplatelet therapy, statin therapy, and risk factor optimization, was associated with favorable clinical improvement. Clinicians should remain aware of such atypical presentations, as timely recognition and imaging-based diagnosis are key to improving outcomes in brainstem strokes.
